# 18F-Fluorodeoxyglucose-Avid Benign Anthracotic Mediastinal Lymphadenitis Mimicking Metastatic Lymphadenopathy

**DOI:** 10.7759/cureus.59614

**Published:** 2024-05-03

**Authors:** Suprabhat Giri, Lohith Kumar, Kiran Mane, Megha S Uppin, Sukanya Bhrugumalla, Sridhar Sundaram

**Affiliations:** 1 Department of Gastroenterology, Nizam's Institute of Medical Sciences, Hyderabad, IND; 2 Department of Digestive Disease and Clinical Nutrition, Tata Memorial Hospital, Mumbai, IND; 3 Department of Pathology, Nizam's Institute of Medical Sciences, Hyderabad, IND

**Keywords:** mediastinal lymph nodes, 18-fdg pet-ct, fine needle biopsy, endoscopic ultrasound, anthracosis

## Abstract

The staging of malignancy is critical for its effective management. 18F-fluorodeoxyglucose (FDG) positron emission tomography-computed tomography (PET/CT) imaging is a common modality for malignancy staging, which identifies areas of FDG avidity. However, multiple benign etiologies can cause false-positive 18F FDG-avid nodes. Among these, extrapulmonary involvement of anthracosis in the form of lymphadenopathy is a rare entity. In patients with concomitant malignancies, the presence of 18F FDG-avid anthracotic lymph nodal enlargement may mimic nodal metastasis. Endosonography-guided tissue acquisition may help differentiate between the two. Herein, we describe six cases of FDG-avid benign anthracotic lymphadenitis detected during staging workups for patients with malignancies who later underwent curative resection.

## Introduction

18F-fluorodeoxyglucose (FDG) positron emission tomography-computed tomography (PET/CT) is increasingly used for staging newly diagnosed malignancy, post-therapy restaging, and long-term surveillance for recurrence. Due to its high contrast lesion-to-background ratio, it is recommended for evaluating non-small cell lung carcinoma (NSCLC), as well as head, neck, and esophageal cancers [1,2]. FDG PET/CT also plays a prognostic role in hematologic malignancies like leukemia, lymphoma, and multiple myeloma, where lymph node involvement is prevalent [3-6]. Apart from primary lymphoid malignancies and metastasis, benign conditions can also present with FDG-avid mediastinal and abdominal lymphadenopathy, mimicking metastatic lymph nodes in patients with concomitant malignancies. Consequently, recent data have endorsed multimodal investigation for accurate staging and evaluation of suspected malignancies [7-9].

Benign conditions that can present as false-positive FDG-avid nodes include mycobacterial and fungal infections, inflammatory conditions like sarcoidosis, and, rarely, anthracosis [10]. Anthracosis, caused by the accumulation of carbon in the lungs from repeated inhalation of smoke or coal dust, primarily involves the pulmonary parenchyma and airways [11] but can also affect extrapulmonary structures, including mediastinal and axillary lymph nodes, paratracheal masses, and the esophagus, presenting as FDG-avid lesions [12]. Therefore, lymphadenopathy in patients with primary malignancies requires biopsy to differentiate malignant from benign etiologies, improving the chances of a patient getting curative therapy.

Here, we report a case series of patients with FDG-avid lymph nodes detected during staging for primary malignancies at different sites, which were later diagnosed as benign anthracotic lymphadenitis (BAL).

## Materials and methods

This retrospective study involved patients who underwent endoscopic ultrasound (EUS)-guided fine-needle aspiration (FNA) or biopsy (FNB) at two tertiary healthcare facilities in India between January 2020 and January 2023. Before the EUS-FNA/B procedures, written informed consent was obtained from each patient. The institutional ethics committee waived the review, and the study was conducted in accordance with the Declaration of Helsinki.

Patient selection

Data from all patients aged 18-80 years who underwent EUS-guided tissue sampling from lymph nodes (mediastinal and abdominal) were screened. Among these, only patients diagnosed with benign anthracotic lymphadenitis alongside concomitant malignancy were included. Demographic parameters, clinical features, imaging and endoscopic findings, and histopathology results were recorded in Microsoft Excel (Microsoft Corporation, Redmond, Washington). The software used for calculating SUVmax was syngo.via (Siemens, Orlando, Florida).

Procedural techniques

EUS was performed by three experienced endosonologists under moderate sedation using midazolam and pentazocine. An oblique-viewing linear echoendoscope (GF-UCT 180 with an Olympus EU-ME2 processor) was used for all procedures. After identifying the lesion and surrounding vasculature using color Doppler, the lesion was punctured in real-time view. 22-G FNA needles (Echotip Ultra, Cook Medical, Bloomington, Indiana) or 22-G FNB needles (Acquire needle®, Boston Scientific Corporation, Natick, Massachusetts) were employed. The number of passes and the suction technique were determined at the endoscopist's discretion [13]. Subsequently, the samples were sent for cytological and histopathological examination [14].

## Results

**Figure 1:**
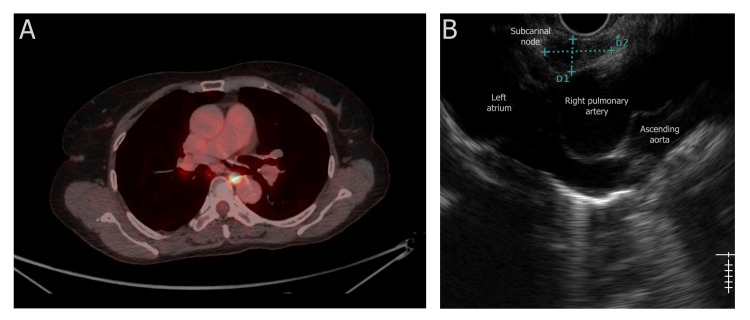
(A) 18F-fluorodeoxyglucose uptake noted in the subcarinal node on PET/CT; (B) endoscopic ultrasound image showing a hypoechoic, well-defined lymph node in station 7

**Figure 2:**
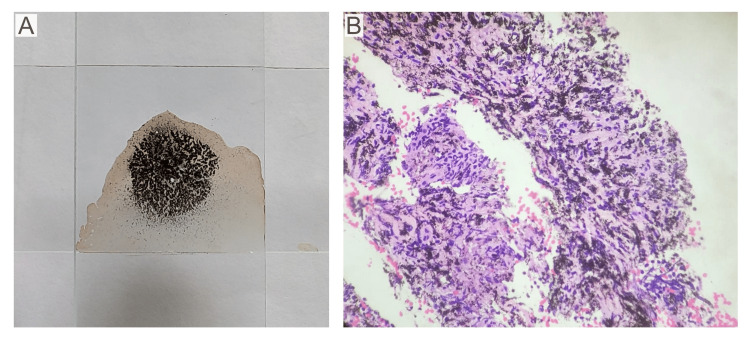
(A) Macroscopic appearance of the tarry black aspirate pressed between two glass slides; (B) microscopic examination showing anthracotic pigment deposition in a background of lymphoid hyperplasia

A total of six cases were diagnosed as anthracosis following EUS-FNA/FNB of FDG-avid lymph nodes detected during staging for malignancies. Table 1 summarizes the patients' characteristics. The median age of the patients was 61.5 years (52-76), with a male-to-female ratio of 1:1. The primary sites of malignancies included the mandibular alveolus, esophagus, ampulla of Vater, pancreas, and gallbladder. All patients underwent PET/CT for malignancy staging and were found to have FDG-avid nodes. All identified lymph nodes were mediastinal, with the subcarinal location being the most common site. The size of the lymph nodes varied from 13 mm to 20 mm along the longest axis. Five cases underwent FNB, while one patient underwent FNA, with a 22-G needle size used in all cases. Figures 1, 2 depict the imaging and histological findings of case 6. Among the six patients, five had a history of exposure to fumes from biomass fuel burning, coal dust, or tobacco smoking. Following the diagnosis of benign anthracotic lymphadenitis (BAL), all patients were eligible for surgical resection.

**Table 1:** Details of the patients with benign anthracotic lymphadenitis SCC: squamous cell carcinoma; NET: neuroendocrine tumor; FNA: fine needle aspiration; FNB: fine needle biopsy

Case	Age/sex	Primary malignancy	Site of FNA from lymph node	Single/multiple lymph nodes	SUV_max_ of node	Size of largest node, in mm	Needle used	Exposure history
Case 1	52/M	Ampullary adenocarcinoma	Subcarinal	Multiple	6.77	13 x 20	22-G FNB	No
Case 2	76/M	SCC of mandibular alveolus	Aortopulmonary	Single	5.1	10 x 19	22-G FNB	Chronic smoking
Case 3	57/M	Gallbladder carcinoma	Subcarinal	Multiple	7.3	10 x 13	22-G FNB	Coal mine
Case 4	72/F	Pancreatic adenocarcinoma	Aortopulmonary	Multiple	4.9	12 x 17	22-G FNB	Biomass fuel
Case 5	63/F	Pancreatic NET	Subcarinal	Multiple	5.63	10 x 16	22-G FNB	Biomass fuel
Case 6	60/F	SCC of esophagus	Subcarinal	Single	4.85	11 x 18	22-G FNA	Biomass fuel

## Discussion

Reports on anthracosis mimicking mediastinal lymph node metastases are scarce. Anthracotic material could provide persistent antigenic stimulation to macrophages, resulting in inflammatory activity highlighted on PET/CT. The present case series describes six cases of BAL detected during the staging workup in patients with malignancies, who were subsequently diagnosed via EUS-FNA/B. In one case report, a patient with adenocarcinoma of the prostate undergoing PET with radiolabelled choline (18F-choline) for pre-treatment staging showed significant uptake in the mediastinal node. Surgical excision of the lymph node followed by histopathology confirmed the presence of anthracosis [15]. In another case, a patient being evaluated for a left upper-lobe lung nodule had FDG-avid bilateral hilar and mediastinal lymph nodes, which were subsequently diagnosed as nodal anthracosis on endobronchial ultrasound-guided FNA [16]. Ivanick et al. reported a case series of patients referred for EBUS-guided biopsies from 18-F FDG PET-positive mediastinal and hilar lymph nodes detected during the workup or treatment for suspected malignancies [17].

Alzubi et al. noted significant exposure to biomass fuels used for cooking in one case of nodal anthracosis [16]. In the study by Ivanick et al., among the 20 patients diagnosed with anthracotic lymphadenitis, the exposure history included dust from outdoor occupations in construction, coal dust from mines, and indoor cooking using biomass fuels [17]. In a previous study on the etiology of mediastinal lymphadenopathy from India, anthracosis was reported in 5% of the cases [10]. Of the 17 patients with anthracosis, two cases had stain or culture positivity for *Mycobacterium tuberculosis*. The remaining 15 patients had endobronchial deposition of black anthracotic pigment on bronchoscopy. However, none of these cases were associated with other malignancies. Concerning the associated conditions with anthracosis, one study reported an association of anthracosis with biomass fuel exposure, stone mining, and pulmonary tuberculosis [18]. Hence, the findings of the present study are critical to tropical countries, where the prevalence of tuberculosis and the use of biomass fuel in rural areas remain high. It is crucial to rule out anthracosis as a cause of FDG-positive nodes in patients with malignancies and significant exposure history.

Anthracosis often presents with radiological features similar to tuberculosis, leading to instances where patients are treated with antitubercular therapy before a definitive diagnosis is established. EUS- or EBUS-guided tissue acquisition allows for differentiation between the two pathologies, thus preventing unnecessary treatment [10]. Kirchner et al. compared CT features between enlarged mediastinal lymph nodes due to anthracosis and malignancy [19]. Features such as larger nodes, ill-defined nodes, contrast enhancement, and necrosis were indicative of a malignant etiology. However, less than 50% of patients with malignant nodes exhibited these features, indicating that cross-sectional imaging may not always successfully differentiate benign nodal anthracosis, thereby necessitating tissue diagnosis.

Despite providing valuable data on a rare entity, this study has a few limitations, including a small sample size and its retrospective design. Additionally, we could not analyze the SUVmax cutoff, which might help differentiate benign from malignant lymphadenopathy. Lastly, we were unable to assess the prevalence of BAL among all patients undergoing PET-CT for staging of various malignancies.

## Conclusions

To conclude, in patients with malignancies undergoing work-up, BAL may be an underrecognized cause of PET-positive lymph nodes. The majority of patients with biopsy-proven BAL typically had a history of inhaling biomass or coal dust. All patients with malignancies presenting FDG-avid nodes should undergo sampling to rule out benign causes of lymph node enlargement, which may provide an opportunity for curative therapy.
